# Impact of Psychotropic Medication Effects on Obesity and the Metabolic Syndrome in People With Serious Mental Illness

**DOI:** 10.3389/fendo.2020.573479

**Published:** 2020-10-09

**Authors:** Victor Mazereel, Johan Detraux, Davy Vancampfort, Ruud van Winkel, Marc De Hert

**Affiliations:** ^1^ Department of Neurosciences, Center for Clinical Psychiatry, KU Leuven, Leuven, Belgium; ^2^ University Psychiatric Center, KU Leuven, Kortenberg, Belgium; ^3^ Department of Rehabilitation Sciences, KU Leuven, Leuven, Belgium; ^4^ Antwerp Health Law and Ethics Chair, AHLEC University Antwerpen, Antwerp, Belgium

**Keywords:** obesity, mood stabilizer, metabolic syndrome, serious mental illness, antipsychotic, antidepressant****

## Abstract

People with serious mental illness (SMI), including schizophrenia, bipolar disorder, and major depressive disorder, have a higher mortality rate and shortened life expectancy. This is mainly attributable to physical diseases, particularly cardiovascular diseases (CVDs). Important risk factors for CVDs are obesity and other metabolic abnormalities, which are especially prevalent in people with SMI. Several factors contribute to this increased risk, including unhealthy lifestyles. Psychotropic medication independently further increases this risk. In this review we want to examine the relationship between obesity and other components of the metabolic syndrome and psychotropic medication in people with SMI.

## Introduction

People with serious mental illness (SMI), including schizophrenia, bipolar disorder (BD), and major depressive disorder (MDD), have a two to three times higher mortality rate than the general population and a 10–20 years reduced life expectancy that appears to be widening (1–9). The majority of deaths in persons with SMI are due to physical diseases, predominantly cardiovascular diseases (CVDs) (10, 11). Despite major advances in cardiovascular health promotion and disease prevention during the last decade, current evidence suggests a limited improvement of CVD risk factors in SMI patients (12). Although a genetic burden, inducing accelerated aging, partly explains the observed premature mortality from physical diseases (9), tackling modifiable CVD risk factors could extend life expectancy.

Obesity and other metabolic abnormalities are important risk factors for CVDs. Obesity is a worldwide problem with estimates of overweight and obesity of 36.9% for men and 38% for women around the globe (13). Compared with people with a body mass index (BMI) of 22.5–25 kg/m², the median survival is reduced by 2–4 years for people with a BMI of 30–35 kg/m² and by 8–10 years for those with a BMI of 40–45 kg/m² (14).

Metabolic abnormalities are especially prevalent in people with SMI. Genetic (15) and non-medical factors, including unhealthy lifestyles and disparities in the health care (6, 7), contribute to this increased prevalence. However, the use of psychotropic medication (antipsychotics, antidepressants and mood stabilizers) can further increase the risk of metabolic abnormalities in these patients (1, 11). Here we will give an overview of the relationship between obesity and other components of the metabolic syndrome and psychotropic medication in people with SMI.

## Obesity

People with SMI are more likely to have overweight or to develop obesity, compared to people who don’t have a mental illness (1, 11, 16). The risk for obesity can be more than four times higher in patients with schizophrenia and about one and a half times higher in those with MDD or BD, compared to the general population (1). Overall, the proportion of SMI patients with abdominal obesity lies between 50 and 63%, depending on the employed criteria (16). Lifestyle factors, such as physical inactivity and an unhealthy diet, as well as psychotropic medication contribute to obesity in these patients (17).

### Effect of Psychotropic Medication on Obesity

Weight gain (at least 7% body weight gain from baseline) (13) is a well-established side-effect of almost all antipsychotics (1, 18). Antipsychotic medications, however, differ in their weight gain liability (11, 18–20) (**Table 1**). The second-generation antipsychotics (SGAs) clozapine and olanzapine appear to have the greatest potential to induce weight gain. Quetiapine, risperidone, paliperidone and iloperidone are associated with a moderate risk for weight gain. Aripiprazole, amisulpride, ziprasidone, asenapine and lurasidone have less or little effect on body weight. Among the first-generation antipsychotics (FGAs) chlorpromazine and thioridazine are reported to induce more weight gain than haloperidol. No antipsychotic should be considered truly weight neutral, as virtually all antipsychotics are associated with weight gain after prolonged use, compared with placebo (1, 11, 18, 19, 21, 22). However, substantial differences exist between individuals in their susceptibility to gain or lose weight with the same antipsychotic (18, 23).

**Table 1:** Impact of psychotropic medication on obesity and other metabolic abnormalities in people with SMI.

*Condition*	*Antipsychotics*	*Antidepressants*	*Mood stabilizers*
*Obesity*	0/+ (for haloperidol, lurasidone, ziprasidone, amisulpride, asenapine, aripiprazole) to +++ (for clozapine, olanzapine, low potency FGAs)	− (bupropion) to + (mirtazapine, paroxetine, TCAs)	0/− (lamotrigine, topiramate) to ++ (lithium, valproate)
*Dyslipidemia*	0/− (cariprazine, aripiprazole, brexpiprazole) to + (clozapine, olanzapine, risperidone, quetiapine)	0 to + (if weight gain)	−/0 (valproate: reduction in cholesterol, increase in triglycerides) to + (lithium)
*Hypertension*	0 to + (if weight gain)	0 (SSRIs) to + (SNRIs, bupropion and TCAs)	0
*Diabetes*	0/+ (for lurasidone, ziprasidone, amisulpride, aripiprazole) to +++ (for clozapine, olanzapine)	−/0 (SSRIs) to ++ (TCAs)	−/0 (lithium, lamotrigine, oxcarbazepine) to + (valproate)

−, reduction; 0, generally no effect; +, some effect; ++, moderate effect; +++, marked effect; ?, uncertain.

FGA, first-generation antipsychotics; TCAs, tricyclic antidepressants; SSRIs, selective serotonin reuptake inhibitors; SNRIs, serotonine and norepinephrine reuptake inhibitors. Table adapted and updated from Correll (1) with permission of the authors.

Compared to antipsychotics, weight gain with antidepressants is generally more modest or mild, and differences between antidepressants are small (1, 24) (**Table 1**). Considering antidepressants in patients with MDD, long-term use (>6 months) and polypharmacy of antidepressants, such as the tricyclic antidepressant (TCA) amitriptyline, the tetracyclic mirtazapine and the selective serotonin reuptake inhibitor (SSRI) paroxetine have been associated with weight gain of up to 2.7 kg (25–28). Bupropion is more likely to cause weight loss than gain (−1.9kg) (27–29). Nevertheless, the effect of each antidepressant on weight gain can vary across individuals, especially in the long term.

The effect of mood stabilizers on weight gain in BD patients is significant, but also to a lesser degree than with the use of antipsychotics (1, 30) (**Table 1**). Weight gain is more frequent with lithium than placebo (OR = 1.89) (31), with a reported 77% of patients experiencing an average weight gain of 4–6.3 kg (32, 33). The most drastic weight changes with lithium occur during the first two years, with higher body weight at the start predicting higher weight increase (33). This is believed to result from increased appetite, hypothyroidism and nephrogenic diabetes insipidus leading to increased thirst (34). Considering antiepileptic mood stabilizers, the use of valproate is associated with weight gain in up to 50% of patients and can be detected 2–3 months after initiation (35). Patients gain on average 6.4 kg (32). This finding does not appear to be dose dependent (36, 37). Carbamazepine has a lower risk of weight gain (38). Lamotrigine at higher doses and topiramate are associated with weight loss of up to 1.2 kg (32, 35). There is currently little evidence supporting the efficacy of combining metformin with a mood stabilizing treatment on weight loss (35).

#### Effects of Psychotropic Medications by Age

A greater magnitude of weight gain following exposure to antipsychotic medication has been reported in children and adolescents (<18 years old) (39). The rank order of weight gain liability of antipsychotics in this vulnerable population remains roughly the same. Olanzapine has been associated with the highest risk of significant weight gain. Risperidone has a somewhat lower tendency to cause weight gain, while aripiprazole has a minor impact on body weight (1, 40–43). The extent of weight gain is also higher in first-episode patients (18, 22), with a mean weight gain of 3.22 kg during short term (<12 weeks) and 5.30 kg during long-term (>12 weeks) treatment (22). Once again, olanzapine seems to induce significantly more long-term (>12 weeks) weight gain in these patients, compared to other antipsychotic medications (22).

In the BD pediatric population a similar trend with mood stabilizers is apparent as in the adult population, however, with less weight gain than antipsychotic therapy. Mood stabilizers lead to an average increase of 1.2 kg over 12 weeks (44).

#### Effects of Psychotropic Medications Over Time

People with SMI often quickly gain weight during the first few weeks of antipsychotic treatment. This rapid increase in weight then gradually decreases and flattens within one year. Initial rapid weight gain is a good predictor for significant long-term weight gain and other metabolic abnormalities. A cohort study with 170 first-episode psychosis patients reported a total mean weight gain of 12.1 kg three years after the initiation of treatment (45). Eighty-five percent of the total weight gain occurs in the first treatment year (30). Switching to an antipsychotic with a lesser tendency to cause weight gain can be an effective intervention for some, but not all patients (18).

### Mechanisms of Medication-Induced Weight Gain

Antipsychotic-induced weight gain/obesity probably occurs as a result of increasing appetite and food intake, as well as delayed satiety signaling. Antagonism at the serotoninergic 5-HT2C and histaminergic H1 receptors has been identified as the key mechanism contributing to this side-effect. Clozapine and olanzapine, having the highest weight gain liability, also have a very strong binding affinity to both 5-HT2C and H1 receptors (21). H1 antagonism has been identified as the strongest predictor of weight gain with antidepressants (31).

A decrease in caloric expenditure due to the sedative effects, and an increased intake of caloric beverages due to dry mouth/throat induced by certain antipsychotics may also contribute to antipsychotic-induced weight gain.

Genetic factors play an important role in medication-induced weight gain, to be estimated at 60-80% for antipsychotic-related weight gain (32). This might help explain the interindividual variability in weight gain to psychotropic medication. In the field of pharmacogenetics, findings suggest the involvement of polymorphisms in several genes coding for proteins implicated in hypothalamic control of food intake and body weight, such as 5HT2C, dopamine D2 receptor, BDNF, insulin-induced gene, melanocortin 4 receptor, synaptosomal-associated protein, leptin, ghrelin and mitochondrial genes (21, 33, 34).

### Management of Medication-Induced Weight Gain

Individual lifestyle counseling, exercise interventions, psychoeducation, and augmentation with aripiprazole or topiramate are considered evidence-based options for the management of antipsychotic-induced weight gain (35). Recent meta-analytical evidence also shows that metformin might decrease weight in adults, adolescents, and children treated with SGAs, although additional high-quality evidence is needed. Moreover, treatment effects with metformin are small (around -3.2 kg over 12–16 weeks) and optimal dosage is unclear (36–38).

### Obesity, Genetics and Environmental Factors

The complex interplay of genetic and lifestyle risk factors influence metabolic disease risk (44). Genome-wide association studies (GWAS) can successfully identify genetic associations with highly polygenic phenotypes in sufficiently large samples (46). Peters et al. (24) identified 40 loci which may contribute to genetic overlaps between mental disorders/traits and BMI and/or shape related phenotypes. Although a recent study of combined GWAS data from 1,380,284 individuals demonstrated an extensive genetic overlap between BMI and SMI, a different genetic liability to weight gain across these disorders seems to exist. While environmental causes of weight gain seem to be more important than disease-specific genetics in patients with schizophrenia, in patients with MDD or BD results point to genetic susceptibility as a more likely cause of weight gain. These finding suggest that factors such as medication, diet, or lifestyle may be the main drivers of weight gain in patient with schizophrenia (25).

### Effect of Obesity and Other Metabolic Abnormalities on Response to Psychotropic Medication

Studies have shown that the presence of obesity or other metabolic abnormalities significantly affects treatment response outcomes in people with SMI. A 1-year naturalistic study by Woo et al. (26) found that the presence of obesity in patients with MDD was associated with a decreased treatment response to antidepressants (OR = 1.55). Males with concurrent metabolic problems (*i.e.* the presence of one or more of hypertension, hyperglycemia, or hypercholesterolemia) had an even higher risk for insufficient response (OR = 2.32). Benedetti et al. (27) showed that BMI can indirectly hamper antidepressant response by increasing the levels of proinflammatory cytokines in patients with BD. This effect of BMI, however, was revealed only when considering cytokines.

## Metabolic Syndrome

The metabolic syndrome (MetS) refers to the clustering of several cardiometabolic conditions, including abdominal obesity, glucose intolerance or insulin resistance, dyslipidemia [higher levels of triglycerides and decreased high-density lipoprotein (HDL) cholesterol levels], and hypertension (11). Several meta-analyses demonstrated that, compared with matched general population controls, people with SMI have a significantly increased risk for developing MetS. When pooling across SMIs, one in three has MetS and no significant difference between diagnostic categories of schizophrenia, BD and MDD can be found (16, 28, 47).

Patients with schizophrenia are 2.35 times more likely to have MetS, compared to the general population, with a prevalence of 34.2% in multi-episode patients. MetS prevalence is significantly lower in drug-naïve (10%) or first-episode patients (15.9%), even after correcting for age (p < 0.001) (29, 48). Patients with BD are 1.98 times more likely to have MetS, with a prevalence rate of 37.4%. BD patients taking antipsychotics have a higher chance of developing MetS than those who are antipsychotic free (OR = 1.72) (49). The increased risk for MetS, however, extends to drug-naïve patients too (50). Looking at MetS risk according to type of antipsychotic, we see a similar pattern as the risk for obesity with clozapine (prevalence rate = 47.2%), quetiapine (prevalence rate = 37.3%) and olanzapine (prevalence rate = 36.2%) being associated with the highest, and aripiprazole (prevalence rate = 19.4%) and amisulpride (prevalence rate = 22.8%) with the lowest MetS risk (16). A recent review of systematic reviews showed that evidence is inconsistent regarding the metabolic side-effects of antipsychotic polypharmacy with some showing no difference to monotherapy and others showing a reduction in weight when combining with aripiprazole (51).

The overall proportion of MDD patients having MetS is 30.5% with an OR of 1.54, when compared to control population (52). One factor contributing to this relationship are the depressive symptoms themselves, especially when vegetative symptoms are present (53, 54). Another factor might be antidepressant use, independent of depressive symptom severity, although not all studies confirm this finding (52, 55–58) (**Table 1**). Moreover, it is still unclear whether the potential increased risk associated with antidepressant use is caused by a single antidepressant class or is a common finding across classes. A recent review pointed to antidepressants with H1-receptor antagonist function to be the main culprit (53). As antipsychotics are often prescribed as augmentation strategy in the treatment of depression, they should be considered as a significant driver for MetS in this group as well (52).

## Dyslipidemia

A meta-analysis showed that patients with SMI have a significantly increased risk for elevated triglycerides (RR = 1.49) and decreased HDL-cholesterol (RR = 1.33), compared to population controls (16). When compared with matched population controls, schizophrenia patients have a 2.73 times higher risk to have hypertriglyceridemia and a 2.35 times higher risk to have reduced HDL-cholesterol levels. In particular patients with multi-episode schizophrenia meet criteria for elevated triglycerides and low HDL-cholesterol more often than first-episode or untreated patients (respectively 39 *vs*. 10.5 and 23.3% for triglycerides and 41.7 *vs*. 16 and 24.2% for low HDL-cholesterol) (29). Interestingly, drug naïve first-episode patients also have dyslipidemia with a systematic review reporting lower levels of HDL and higher levels of triglycerides, compared to controls (59). A GWAS revealed that the levels of LDL- and HDL-cholesterol together with schizophrenia risk share common genetic risk factors (60).

A similar picture arises in patients with BD having higher total and LDL- and lower HDL-cholesterol levels (16, 61). Moreover, no significant differences seem to exist between schizophrenia and BD patients regarding HDL-cholesterol and triglycerides (28). Again, these abnormalities may already be present in drug-naïve BD patients (62). The lipid abnormalities in BD may be state dependent with depression resulting in more anomalous levels than mania (63).

A higher risk for hypertriglyceridemia (but not lower HDL-cholesterol) was also found in patients with MDD, compared with age- and gender-matched general population controls (52). However, in another study with BD and MDD patients lower HDL- and higher total and LDL-cholesterol was found during the depressive episode (61). This suggests that these lipid abnormalities might again be state dependent. Just as with schizophrenia, mood disorders and metabolic abnormalities might share common genetic risk factors (64).

Although dyslipidemia may precede the use of medication in patients with SMI, there is sufficient evidence showing that certain types of psychotropic medication can independently lead to further abnormalities (**Table 1**). Antipsychotics have been associated with lipid abnormalities (10, 23). Levels of triglycerides and cholesterol can increase during the early stage of antipsychotic treatment. These adverse effects, however, may even precede antipsychotic-induced weight gain, pointing to weight-independent molecular effects, in addition to weight-related ones (23). Clozapine, olanzapine and risperidone have all been associated with a higher chance of mild dyslipidemia, compared with FGAs such as haloperidol, although differences are small and one should focus more on the metabolic profile of each individual antipsychotic (65). A recent network meta-analysis showed that the increased total cholesterol with clozapine, olanzapine and quetiapine was moderated by race, with non-white participants having larger increases. Cariprazine decreased LDL-cholesterol, quetiapine and olanzapine increased LDL-cholesterol, brexpiprazole and aripiprazole increased HDL-cholesterol, and quetiapine, olanzapine, clozapine and zotepine increased triglycerides (20). Regarding antipsychotic polypharmacy, a systematic review reported better lipid profiles when the augmentation drug of choice was aripiprazole (51). This might therefore be a viable strategy to attenuate the detrimental effects of certain antipsychotics on lipid profiles.

Treatment with statins in schizophrenia patients decreases LDL-cholesterol, total cholesterol and triglycerides, but does not alter HDL-cholesterol (66–68). Other efficacious interventions for dyslipidemia are topiramate for LDL-cholesterol and triglycerides, and lifestyle interventions for total cholesterol, LDL-cholesterol and triglycerides (35).

Among mood stabilizers, lithium has not been associated with clinically relevant lipid abnormalities besides a mildly elevated increase in triglycerides (69). However, lithium-induced hypothyroidism can lead to weight gain and changes in lipid profile. Despite its association with weight gain and increased triglycerides, valproate has been correlated with reductions in total and LDL-cholesterol in patients with schizophrenia and BD (1). In children and adolescents with BD, valproate does not appear to increase the risk of dyslipidemia and may in fact improve lipid profiles to some degree (70, 71). Carbamazepine is associated with a similar increase in both HDL- and LDL-cholesterol (72).

Although some antidepressants have been associated with weight gain, which is a risk factor for lipid abnormalities, data on adverse lipid effects with these medications remain scarce. At this moment, results show that most antidepressants have not been associated with dyslipidemia. Moreover, a direct weight-independent effect on serum cholesterol has not been consistently reported (1). When combining a SSRI with olanzapine, quetiapine or risperidone in patients with schizophrenia or BD a negligible increase is seen in total and LDL-cholesterol and triglycerides (73, 74).

## Hypertension

A meta-analysis found that, compared with matched general population controls, people with SMI do not seem to have a significantly increased risk for hypertension (p = 0.07) (16, 52). However, another recent meta-analysis in BD and schizophrenia patients did report an increased risk for hypertension in BD patients (Incidence Rate Ratio = 1.27) (75). A recent large representative cohort study in Taiwan showed that schizophrenia patients were 1.93 times more likely to be diagnosed with hypertension in the year before schizophrenia was diagnosed (76). A possible explanation for the disparity in results is that blood pressure in BD, schizophrenia and MDD patients is poorly recorded and that this may have led previous studies to underestimate the risk of hypertension in SMI patients (75, 77).

The literature on the appearance of hypertension due to antipsychotics seems to be restricted to case studies/series (78, 79). The largest existing cohort study, comprising 284,234 individuals, showed that those with 1 year of exposure to SGAs showed only a small heightened risk of essential hypertension (Hazard Ratio, HR = 1.16, p < 0.0001), compared to those using antidepressants (80) (**Table 1**). Moreover, there does not seem to be an additive effect on blood pressure when combining antipsychotics (51). The possible small increased risk in hypertension after antipsychotic treatment probably can be attributed to antipsychotic-induced weight gain/obesity (81) and to the anti-dopaminergic effect of these agents, as all 5 dopamine receptor subtypes (D1–D5) regulate blood pressure (78, 82).

Generally, mood stabilizers do not affect blood pressure, unless chronic renal failure induced by lithium affects volume distribution (1) or these agents cause weight gain (**Table 1**). Carbamazepine has been associated with acute onset of hypertension in several case reports (83–85). It should be noted that concomitant use of angiotensin-converting enzyme (ACE)-inhibitors or angiotensin II receptor blockers (ARBs) with lithium can lead to acute or chronic lithium toxicity (86, 87).

Antidepressants also are associated with only a small increased risk for hypertension (HR = 1.16, p < 0.001) (88) (**Table 1**). Among antidepressants, TCAs (mainly attributed to the anticholinergic effects of these agents) and serotonin-norepinephrin reuptake inhibitors (SNRIs), particularly venlafaxine, show a significantly higher risk for hypertension with increases up to 15 mmHg, while SSRIs do not affect blood pressure (1, 89–91). In addition, bupropion has been shown to increase blood pressure up to 7.5mmHg. The combination bupropion-naltrexone (for the treatment of obesity) also mildly increases blood pressure (81, 92).

## Diabetes Mellitus

The prevalence of type 2 diabetes mellitus (DM) in people with SMI is estimated at 11.3%, which is 2–3 fold higher than in the general population (1, 29, 93, 94). This figure, however, might even be an underestimate since up to 70% of SMI patients with DM remain undiagnosed (95–97). There seems to be no difference in DM prevalence rates between schizophrenia, BD or MDD. Multi-episode schizophrenia confers a higher risk for DM than first-episode psychosis. Women also are at higher risk of developing DM (94). Compared with the general population, the onset of DM can occur about 10–20 years earlier in individuals with schizophrenia and BD (93).

### Effect of Psychotropic Medication on DM

Although an association seems to exist between antipsychotic medication and DM, further research is needed to understand how these medications cause DM (93, 98). The estimated prevalence of DM in antipsychotic-naïve patients is 2.9%, which is substantially lower than in patients treated with antipsychotics except for aripiprazole and amisulpride. Treatment duration is associated with higher prevalence rates (94). Particularly olanzapine and clozapine have been linked with a higher risk of glucose dysregulation or DM in people with schizophrenia or BD, followed in ranking by asenapine, paliperidone, quetiapine and risperidone (1, 93, 99, 100). Ziprasidone, lurasidone and aripiprazole are associated with minimal glucose changes, compared to other antipsychotics or placebo (100). Aripiprazole augmentation can lead to slightly reduced glucose levels and improvement in HbA1c, although these improvements are statistically non-significant (51).

A recent meta-analysis (101) confirmed the association between antidepressant use and new-onset DM (RR = 1.27, p < 0.001) that has been found in previous meta-analyses (102, 103), reporting a 1.3 to 1.5-fold increase of new-onset DM among antidepressant users, compared with non-users (**Table 1**). Whether antidepressant use causes DM is still not firmly established (101, 104). People with MDD have an increased risk of developing DM (101), while reducing depressive symptoms does lead to better glycemic control independently of weight changes (105). Although it is unclear whether certain antidepressants have a different effect on the risk of DM (101), short-term use of SSRIs in general stabilize or lower blood glucose levels (with a possible risk of hypoglycemia), while TCAs are associated with hyperglycemia and worsening of glycemic control (99, 105, 106). Particularly people with MDD who have been treated with high or moderate doses for a long time are at an increased of developing DM (106). Whether the concurrent use of (certain) antidepressants is associated with an increased risk of glucose dysregulation or DM remains to be proven (1, 103, 104).

Considering mood stabilizers, mixed results are observed (**Table 1**). On the one hand, no significant change in glucose metabolism has been associated with lithium in several older studies (69, 107, 108). However, treatment duration can be an important moderator variable as a 0.79% yearly increase in glucose levels with lithium only seemed to start after a 6-10 years treatment period (109). On the other hand, a recent population based study in 565,253 BD patients without prior glucose metabolism-related diagnoses found that lithium was associated with a decreased risk for developing DM, compared to a “no drug” regimen (110). Regarding valproate, little is known about its effects on glucose metabolism in BD patients. In patients with epilepsy, it has been established that valproate leads to hyperinsulinemia and insulin resistance after the initial weight gain (111). However, valproate monotherapy was not associated with increased risk for DM in BD patients in a recent study by Nestsiarovich (110). When looking at other antiepileptic agents, both lamotrigine and oxcarbazepine seem to be associated with a reduced risk for DM in the same patient population (110), which is in accordance with earlier studies showing that they either have a positive or neutral effect on glucose metabolism (112, 113). Taking into account that many patients with BD need a combination of mood stabilizing agents, antipsychotics and antidepressants, it is disconcerting to note that these combinations lead to a 1.07–2.37 higher risk for DM (110).

### Effects of Psychotropic Medication by Age

As antipsychotic-associated DM is clearly a major issue in adults, it emphasizes the need for an even more judicious use of this class of medications in children and adolescents (4, 10, 114). Children who are prescribed a SGA have a 2–3 times higher risk of developing type 2 DM, compared with SGA-naive children. Higher cumulative doses (particularly with olanzapine), longer treatment duration, and adjunctive antidepressant use may increase this risk even further, which seems to remain high for a certain period of time after discontinuation (114–116). The metabolic adverse effects during SGA treatment more than likely are mediated by biological or genetic factors, explaining why some children and adolescents are more susceptible to these adverse effects than others (1, 4).

Children and adolescents who use antidepressants also appear to have an elevated risk for type 2 DM (116, 117). Recent evidence suggests that in antipsychotic-treated youth, concomitant SSRI/SNRI use is associated with an even higher risk of type 2 DM, which markedly intensifies with increasing duration of SSRI/SNRI use and cumulative SSRI/SNRI dose (117, 118).

### Mechanisms of Medication-Induced DM

The potential mechanisms for antipsychotic-induced DM include: (1) antipsychotic-induced insulin resistance through weight gain/obesity, (2) insulin resistance due to direct effects of antipsychotics, and (3) antipsychotic-induced *β*-cell dysfunction and apoptosis (119). Antipsychotics thus appear to contribute to insulin resistance and DM both indirectly, by inducing weight gain, and directly, by promoting insulin resistance and *β*-cell deterioration. M3 receptors play a key role in maintaining proper insulin release through both peripheral and central cholinergic pathways. Olanzapine and clozapine, the SGAs associated with the greatest the risk of DM, also have high binding affinity with M3 receptors (120, 121). Certain genetic polymorphisms can also lead to dysregulated glucose metabolism when combined with antipsychotics (122).

Multiple explanations have been proposed to explain why antidepressant exposure increases the risk of type 2 DM, including antidepressant-induced weight gain, abnormal glucose metabolism through insulin resistance, inhibition of insulin secretion and hyperglycemia, particularly with antidepressants that have a high affinity for the norepinephrine reuptake transporter, serotoninergic 5-HT2C receptor, and histaminergic H1 receptor (118).

## Summary

Although metabolic abnormalities may be already present in drug naïve SMI patients, reflecting disease-specific mechanisms, it appears that the development of these abnormalities is also attributable to a cumulative long-term effect of unhealthy lifestyle choices and psychotropic medication use, independent of psychiatric disease (123–125). In general, metabolic abnormalities are most common with antipsychotics. Antidepressants and mood stabilizers have a less profound impact on metabolic parameters. However, effects vary greatly with different medications, and interactions with underlying individual factors are relevant. The use of high dosages or multiple medications, as well as the treatment of vulnerable populations seem to be associated with more harmful metabolic consequences.

Screening, assessment, and management of metabolic aspects in patients with SMI remain poor, even in developed countries (4, 11, 126). It is therefore imperative that psychiatrists, physical health specialists and general practitioners are aware of the interaction between SMI, psychotropics and metabolic abnormalities and collaborate in the integrated care for these patients so that they can receive optimal treatment. Furthermore, governments, insurance companies, health care provider and research funding bodies can take a central role by focusing on implementation of integrated physical and mental health treatment programs, innovations in digital health technologies and ensuring equitable health care access. A blueprint for this worldwide priority for people with SMI has already been conceptualized (127). It is time to put this research into practice.

## Author Contributions

VM and JD wrote the first draft. DV, RW, and MH commented on the first draft. MH supervised the writing. All authors contributed to the article and approved the submitted version.

## Conflict of Interest

The authors declare that the research was conducted in the absence of any commercial or financial relationships that could be construed as a potential conflict of interest.
